# On-grid and in-flow mixing for time-resolved cryo-EM

**DOI:** 10.1107/S2059798321008810

**Published:** 2021-09-22

**Authors:** David P. Klebl, Howard D. White, Frank Sobott, Stephen P. Muench

**Affiliations:** a School of Biomedical Sciences, University of Leeds, Leeds LS2 9JT, United Kingdom; b Astbury Centre for Structural and Molecular Biology, University of Leeds, Leeds LS2 9JT, United Kingdom; c Department of Physiological Sciences, Eastern Virginia Medical School, Norfolk, Virginia, USA; d School of Molecular and Cellular Biology, University of Leeds, Leeds LS2 9JT, United Kingdom

**Keywords:** electron microscopy, time-resolved, myosin, structural biology, protein dynamics

## Abstract

Time-resolved cryo-electron microscopy (TrEM) allows the study of proteins under non-equilibrium conditions on the millisecond timescale, permitting the analysis of large-scale conformational changes or assembly and disassembly processes. However, the technique is developing and there have been few comparisons with other biochemical kinetic studies. Using current methods, the shortest time delay is on the millisecond timescale (∼5–10 ms), given by the delay between sample application and vitrification, and generating longer time points requires additional approaches such as using a longer delay line between the mixing element and nozzle, or an incubation step on the grid. To compare approaches, the reaction of ATP with the skeletal actomyosin S1 complex was followed on grids prepared with a 7–700 ms delay between mixing and vitrification. Classification of the cryo-EM data allows kinetic information to be derived which agrees with previous biochemical measurements, showing fast dissociation, low occupancy during steady-state hydrolysis and rebinding once ATP has been hydrolysed. However, this rebinding effect is much less pronounced when on-grid mixing is used and may be influenced by interactions with the air–water interface. Moreover, in-flow mixing results in a broader distribution of reaction times due to the range of velocities in a laminar flow profile (temporal spread), especially for longer time delays. This work shows the potential of TrEM, but also highlights challenges and opportunities for further development.

## Abstract 2

Time-resolved cryo-EM allows the study of proteins under non-equilibrium conditions on the millisecond timescale. Here, in-flow and on-grid mixing techniques are directly compared and it is found that on-grid reactions can be influenced by air–water interactions, whilst in-flow reactions give a broader distribution of reaction times due to laminar flow.

## 1. Introduction   

Motor proteins and many other biological macromolecules change conformations, form complexes or dissociate from their interaction partners as part of their functional cycle. Such reactions often involve a series of transient intermediate states which can be trapped and analysed if the system is studied with appropriate temporal resolution. Cryo-electron microscopy (cryo-EM) is well suited for such studies: conformations may be separated *in silico* if they are structurally different and present in sufficient number upon vitrification. In principle, conventional grid-making approaches allow time-resolved studies, but typical blotting is inherently slow because of the manual sample application and the relatively long blot times (seconds). This typically limits the technique to time points longer than ∼10 s. With fast grid-preparation methods, however, a reaction may be initiated and quenched with a very short and defined time delay (>5 ms). Unwin first demonstrated this approach of rapid mixing, freeze-quenching and EM image processing to study the active state of the acetyl­choline receptor in the mid-1990s (Unwin, 1995 ▸). More recent advances in biological cryo-EM promise to make this method more broadly applicable. These include software developments to account for continuous flexibility (Nakane *et al.*, 2018 ▸), the improved signal to noise obtained with modern microscope hardware, and developments in fast cryo-EM grid preparation (Feng *et al.*, 2017 ▸; Rubinstein *et al.*, 2019 ▸; Jain *et al.*, 2012 ▸). The reaction is typically initiated by rapid mixing at a defined time point before vitrification. Mixing can either be achieved within custom-built microfluidic devices (Kaledhonkar *et al.*, 2018 ▸; Mäeots *et al.*, 2020 ▸) or on the grid (Berriman & Unwin, 1994 ▸; Dandey *et al.*, 2020 ▸). The reaction then proceeds until it is stopped by vitrification of the sample.

While starting the reaction by rapid mixing is perhaps the most versatile method of reaction initiation, it comes with limitations. In a microfluidic setup the liquid flow within the channels is laminar, resulting in a spread of time points, which affects longer time delays more strongly (Mäeots *et al.*, 2020 ▸). If the reaction is incubated on the grid, more closely resembling a stopped-flow setup, this spread could be eliminated. On the grid, however, particles are confined within the support film and/or air–water interfaces. Interactions, especially with the air–water interface, can result in preferred particle orientation and particle denaturation (Noble, Dandey *et al.*, 2018 ▸; D’Imprima *et al.*, 2019 ▸). These effects of preferred orientation and particle denaturation can occur on a millisecond timescale (Noble, Wei *et al.*, 2018 ▸; Klebl, Gravett *et al.*, 2020 ▸). Especially time points with longer reaction times on-grid may therefore be affected by the air–water or water–support interfaces.

In this work, we compare on-grid and in-flow mixing by following the reaction of ATP and skeletal actomyosin S1 by TrEM to establish which may be most suitable for different time delays. We chose the actomyosin complex as a test system because it was among the first assemblies to be studied by time-resolved cryo-EM (Walker *et al.*, 1995 ▸, 1999 ▸). Its kinetics are well understood from biochemical experiments, and equilibrium structures have been determined for a variety of different actomyosin complexes (von der Ecken *et al.*, 2016 ▸; Fujii & Namba, 2017 ▸; Risi *et al.*, 2021 ▸). As part of its catalytic cycle, the myosin motor alternates between states of high and low affinity for filamentous actin (F-actin). This is necessary to allow the cycle of attachment, force generation and dissociation, for example to achieve stepwise movement in the ‘two-headed’ myosin V (Walker *et al.*, 2000 ▸). In the absence of nucleotide, myosin motors bind F-actin filaments with high affinity. Mixing with ATP leads to ATP binding by the myosin motor, a reduction in affinity and dissociation of the motor from the filament (Millar & Geeves, 1983 ▸). Using fast on-grid mixing time points at 7 and 13 ms, we followed this initial dissociation reaction of skeletal actomyosin S1. The actomyosin system then hydrolyses ATP in the steady state. When most ATP has been turned over, the actomyosin complex reassociates (White & Taylor, 1976 ▸). These steps were followed by slow on-grid mixing at 340 and 640 ms and in-flow mixing at 400 and 700 ms.

## 2. Methods   

### 2.1. Protein preparation   

Monomeric rabbit G-actin was obtained as described previously (Spudich & Watt, 1971 ▸). For polymerization, G-actin was mixed with 10%(*v*/*v*) exchange buffer (3 m*M* MgCl_2_, 11 m*M* EGTA) and incubated on ice for 5 min. 10%(*v*/*v*) polymerization buffer (120 m*M* MOPS, 300 m*M* KCl, 12 m*M* MgCl_2_, 1 m*M* EGTA) was then added and the solution was incubated for at least 2 h on ice to allow polymerization. F-actin was then diluted to the target concentration in reaction buffer (10 m*M* MOPS, 2 m*M* MgCl_2_, 0.1 m*M* EGTA, 50 m*M* potassium acetate pH 7). Rabbit skeletal myosin S1 (A1 fraction) was prepared as described previously (White & Taylor, 1976 ▸). The actomyosin complex was obtained by mixing F-actin and myosin S1 in a 1:1 molar ratio at final concentrations of 40 µ*M* in reaction buffer. Disodium ATP (Roche) was prepared as a 100 m*M* stock solution in water at pH 7, stored at −20°C and diluted in reaction buffer to 200 µ*M* before use.

### 2.2. Time-resolved cryo-EM grid preparation   

All grids were prepared using our in-house setup for time-resolved cryo-EM (Kontziampasis *et al.*, 2019 ▸). The key experimental conditions for TrEM grid-preparation experiments are listed in Tables 1 ▸ and 2 ▸. In all TrEM experiments, 40 µ*M* actomyosin complex was mixed with 200 µ*M* ATP in a 1:1(*v*:*v*) ratio to give final concentrations of 20 µ*M* actomyosin and 100 µ*M* ATP. Sample application was performed using gas dynamic virtual nozzles in spraying mode, as described previously (Klebl, Monteiro *et al.*, 2020 ▸). The spray gas pressure was 2 bar. All grids were prepared at ambient temperature (∼20°C) and at a relative humidity of >60%. For on-grid mixing, three separate syringes were used (Table 1 ▸). The three liquid flows meet in the microfluidic mixer/sprayer just before exiting the spray nozzle. For in-flow mixing, an external T-mixer (Upchurch Micro Static Mixing Tee) was introduced upstream of the nozzle. After passing a delay line (Table 2 ▸), the reaction mixture was sprayed using the same nozzle design as used for on-grid mixing, but only making use of the central channel. Quantifoil 300-mesh Cu R1.2/1.3 grids were used after glow-discharge in air for 90 s at 10 mA and 0.1 mbar using a Cressington 208 carbon coater with a glow-discharge unit.

### 2.3. Estimation of time delays   

For on-grid mixing experiments, the time delay was estimated by measuring the plunge speeds with a linear potentiometer and oscilloscope (Hantek 6022BE) recording at 1 MHz or 500 kHz, as described previously (Kontziampasis *et al.*, 2019 ▸). With a total liquid flow rate of 2–4 µl s^−1^, the dead time between the mixing element and spray nozzle was estimated to be less than 1 ms. Similarly, the delay between spray generation and spray application was less than 1 ms, given the high droplet speed of ≥10 m s^−1^ (Klebl, Monteiro *et al.*, 2020 ▸). Thus, only the plunge speed, the distance between the nozzle and ethane, and stop times (for 340 and 640 ms delays) were considered (Table 1 ▸). The spray cone was approximately 5 mm wide at the point of sample application, leading to an estimated error of 2–3 ms.

For in-flow mixing experiments, total flow rates of 4 µl s^−1^ were used. The volume of the delay line was 0.35 or 2 µl, depending on the tube inner diameter (ID) used (Table 2 ▸). At the given tube diameter and liquid flow rate, the estimated Reynolds number (Re) is between 14 and 34, well below the transition of laminar to turbulent flow at Re ≃ 2000 (Avila *et al.*, 2011 ▸). Thus, the flow is expected to be laminar in the delay line. The residence time distribution *E*(*t*) for a laminar flow reactor, such as the delay line used here, is (Fogler, 2010 ▸)
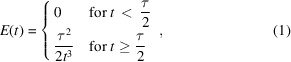
where τ is the mean residence time, given by the tube volume divided by the volumetric flow rate. Under the experimental conditions, diffusion should have a negligible effect on the residence time distribution. The mixer dead volume was 0.95 µl and the spray-nozzle dead volume was estimated as 0.3 µl. The time between sample application and vitrification was 19 and 23 ms, respectively, for these experiments (with plunge speeds of 1.2 and 1.0 m s^−1^ and a nozzle–ethane distance of 2.3 cm). Thus, median residence times of 400 ms (394 ms) and 700 ms (696 ms) were calculated for the 150 and 360 µm ID delay lines, respectively. Considering laminar flow in the delay line, 95% of particles had calculated residence times of 376–622 ms and 593–2016 ms for the 150 and 360 µm ID delay lines, respectively.

### 2.4. Cryo-EM data collection and processing   

All cryo-EM data, except for the 13 ms time point, were collected on a Titan Krios microscope equipped with a Falcon III detector in integrating mode. The 13 ms data were collected on a Titan Krios microscope equipped with a Gatan K2 detector in counting mode. Key data-collection parameters are listed in Table 3 ▸. All image processing was performed using helical and single-particle processing in *RELION* 3.0 (He & Scheres, 2017 ▸; Zivanov *et al.*, 2018 ▸). Micrographs were motion-corrected using *MotionCor*2 (Zheng *et al.*, 2017 ▸) and CTF estimation was performed with *Gctf* (Zhang, 2016 ▸). All filaments were manually picked, extracted and rescaled to a 200-pixel box with a nominal pixel size of 2.13 Å per pixel. For the two different detectors used (Table 3 ▸), the optimal pixel size ratio was determined by cross-correlation of maps and appropriate rescaling during extraction (Wilkinson *et al.*, 2019 ▸). One round of 2D classification was used to exclude poor-quality particles; less than 15% of particles were excluded from each time point. A consensus helical reconstruction was then calculated for each time point. For further processing, all data sets were combined. The processing strategy for the combined data is shown in Supplementary Fig. S1. Focused classification without alignment was used to identify myosin-bound and unbound actin subunits. Unbound actin subunits were selected and used to generate a reconstruction by standard helical processing. For the myosin-bound subunits, signal for the actin filament was subtracted, leaving only a central actin trimer with one myosin molecule bound. These actomyosin particles were then refined as single particles. Finally, the particles were traced back to their original data set (time point) and the per-micrograph distribution was analysed with an in-house Python script. Fits to the data and statistical tests were performed with *GraphPad Prism* 7.

## 3. Results   

**Figure 1:**
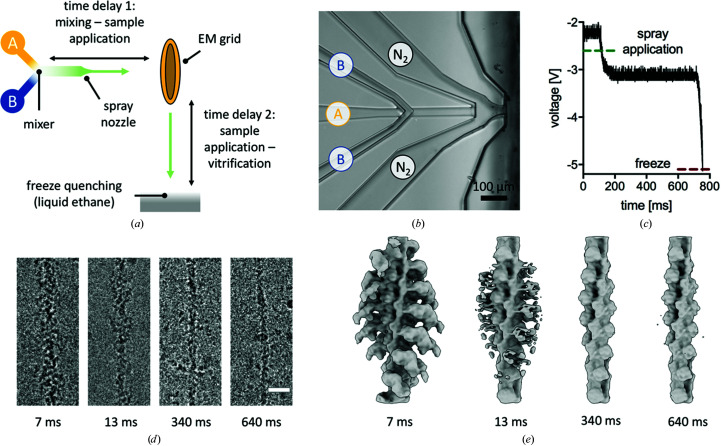
TrEM of actomyosin dissociation. (*a*) Schematic of the TED with different delay times indicated. (*b*) Microscopic image of the microfluidic gas dynamic virtual nozzle with mixing element used in this work. (*c*) Oscilloscope recording for a grid prepared with a stop of 600 ms between spray application and vitrification. The measured voltage corresponds to the vertical position of the grid. (*d*) Raw cryo-EM images of the actomyosin complex mixed with ATP and vitrified after 7, 13, 340 and 640 ms. The scale bar corresponds to 20 nm. (*e*) Consensus reconstructions of the actomyosin complex 7, 13, 340 and 640 ms after on-grid mixing with ATP, all shown at a 3σ threshold.

Two major steps determine the time delay between mixing and freeze-quenching with our custom-built time-resolved EM device (TED) and in most other time-resolved EM devices. These time delays are schematically shown in Fig. 1 ▸(*a*). The first relevant time delay is the time from mixing to sample application. In our current nozzle design (Fig. 1 ▸ *b*) this delay is minimal; we expect mixing to occur in the spray or on-grid. The second time delay is the time between sample application and vitrification. We prepared cryo-EM grids of the skeletal actomyosin complex mixed with ATP, varying the time between sample application and vitrification between 7 and 640 ms (corresponding to time delay 2 in Fig. 1 ▸ *a*). Time delays of 340 and 640 ms were achieved by stopping the pneumatic plunger for a defined time after passing the spray before vitrification (Fig. 1 ▸ *c*). This approach is preferable over a slow continuous motion, because slow immersion in liquid ethane at speeds significantly lower than 1 m s^−1^ results in the formation of crystalline ice (Kasas *et al.*, 2003 ▸). For the short time delays of 7 and 13 ms, grids were plunged without a stop step before final plunging at high speeds (Table 1 ▸).

The resulting cryo-EM images of the actomyosin complex after mixing with ATP show, as expected, time-dependent dissociation of myosin S1 from the filaments (Fig. 1 ▸ *d*). A small cryo-EM data set was collected at each of the four time points (7, 13, 340 and 640 ms) and the images were processed. Like the raw images, the 3D reconstructions show most myosin S1 bound at 7 ms, less myosin bound after 13 ms and very weak myosin density, at thresholds below 3σ, at the 340 and 640 ms time points (Fig. 1 ▸ *e*). While they indicate the course of the reaction, these consensus reconstructions only give limited insight because they are an average of all filaments from the respective data set containing both decorated and undecorated filaments. In order to quantify the particles, we performed focused 3D classification without alignment. Each particle contains one unique, central myosin-binding site because particles were extracted along the filament with an inter-box distance close or equal to the helical rise (27.5 Å). By using a mask for the central myosin site, the myosin occupancy at this site was probed. To impose the same classification criteria and allow better comparison, all data sets were combined for classification. We note that the classification outcome showed approximately 5% variability in relative particle number depending on the parameters used. We also found that the classification was biased towards more pre­dominant states, leading to a similar variability in particle numbers. This provides an estimate of the uncertainty introduced by 3D classification, even with a relatively large difference (myosin S1 present or absent) between states.

**Figure 2:**
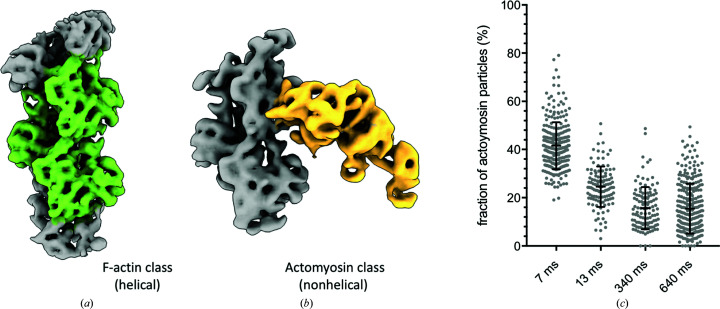
TrEM data processing. Reconstructions of F-actin (*a*) and the actomyosin complex (*b*) from the combined data. The central three actin subunits are shown in green and myosin S1 is in yellow. (*c*) Relative particle numbers of the actomyosin complex traced back to individual time points. Shown is the percentage of actomyosin particles relative to the total number of myosin-binding sites (the number of actin subunits). For each time point, the mean and standard deviation are shown as black lines and per-micrograph particle numbers are shown as grey points.

From the combined data, we obtained a 4.8 Å resolution structure of F-actin and a 7.5 Å resolution structure of the skeletal actomyosin complex (Figs. 2 ▸ *a* and 2 ▸ *b*). While free F-actin was processed by the standard helical method, actomyosin particles were processed with a single-particle approach after subtracting signal for all but the central three actin subunits and the bound myosin. This gave improved density for the radially distant regions of the myosin motor, with only a few particles being misaligned in the absence of helical constraints (Supplementary Fig. S2). The reconstructions (at 4.8 and 7.5 Å resolution, respectively) were the same as the published structures of F-actin and skeletal actomyosin in the rigor state, respectively (Fujii & Namba, 2017 ▸; Merino *et al.*, 2018 ▸). In the actomyosin reconstruction, the actin backbone and myosin motor have similarly strong density, suggesting that this set of particles has very high, or full, myosin occupancy. The data also contained a subset of particles which were not assigned to either class (Supplementary Fig. S2). This population was probably a mixture of unbound and bound states, and potentially other weakly bound states.

We quantified the relative number of particles in the two identified states (bare actin and actomyosin) at each time point by tracing the particles back to their original data set. As expected, there is a decrease in the relative number of actomyosin particles over time, from ∼42% occupancy at 7 ms to ∼16% occupancy at 340 ms (Fig. 2 ▸ *c*). Plotting the per-micrograph relative number of actomyosin particles showed a significant spread between micrographs from a single grid and time point. These results show that ATP-induced dissociation of the actomyosin complex can be quantitatively followed by varying the on-grid delay time. An exponential fit to the on-grid TrEM data gave a pseudo-first-order reaction rate of 177 s^−1^, similar to published values for this reaction (White & Taylor, 1976 ▸). The fraction of actomyosin complex was not significantly different between the 340 and 640 ms time points (*p* = 0.3, Mann–Whitney test), indicating that the reaction was completed or remained in a steady state.

**Figure 3:**
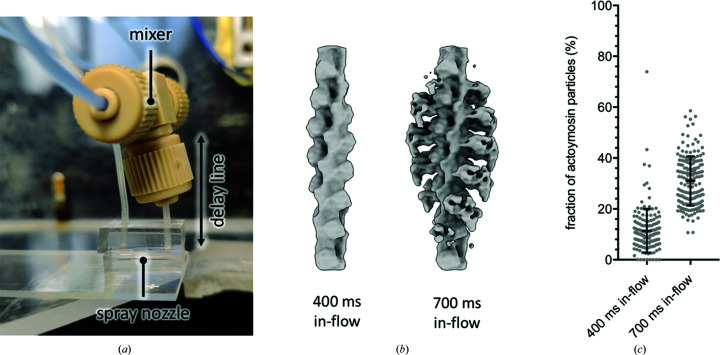
Longer time delays by in-flow mixing. (*a*) Photograph of the experimental setup with T-mixer, delay line and spray nozzle. The delay line length is 2 cm. (*b*) Consensus reconstructions of the actomyosin complex 400 or 700 ms after mixing with ATP in-flow shown at 3σ. (*c*) Relative particle numbers of the actomyosin complex traced back to individual time points. Shown is the fraction of actomyosin particles relative to the total number of myosin-binding sites (the number of actin subunits). For each time point, the mean and standard deviation are shown as black lines and per-micrograph particle numbers are shown as grey points.

The reaction cycle of the actomyosin complex is such that after dissociation, reassociation should occur at longer time delays. However, no reassociation was seen when the mixing occurred predominantly ‘on-grid’. In order to examine whether this may be a feature of on-grid mixing, a different experimental setup was used. An external mixing unit and delay line were introduced upstream of the nozzle (Fig. 3 ▸ *a*). This corresponds to changing time delay 1 in Fig. 1 ▸(*a*): the two reactants meet in a T-mixer, flow through the delay line, are sprayed onto the EM grid and are then vitrified. Using this approach, we could minimize any interactions with the air–water interface which may occur during on-grid mixing due to the length of time that the protein resides on the grid. In this case the delay line ID and liquid flow rate, as well as the mixer and sprayer dead volumes, determined the total time delay. Grids were prepared at two different time points, with median time delays of 400 or 700 ms. We assumed laminar flow in the delay line. In this case the setup resembles a laminar flow reactor (Fogler, 2010 ▸) and the time points are spread by the parabolic flow profile in the delay line; see Section 2 for details. The 400 or 700 ms in-flow time points were analysed in the same way as the on-grid mixing data. Consensus reconstructions from each time point, at a threshold of 3σ, show that little myosin was bound at 400 ms (consistent with the on-grid mixing experiment) but an increase in myosin density for the later 700 ms time point was observed, in contrast to the on-grid mixing approach, where no reassociation is observed at 640 ms (Fig. 3 ▸ *b*).

For classification, the 400 and 700 ms in-flow mixing data were processed with the on-grid data to allow a direct comparison. The 400 ms in-flow data showed the lowest fraction of myosin bound, 11%, and there was an increase in bound myosin particles at the 700 ms time point (Fig. 3 ▸ *c*). This was in contrast to the 640 ms on-grid time point. Such an increase in actin binding is expected when most of the available ATP has been hydrolysed.

## 4. Discussion   

There have been some significant steps in recent years in the development of time-resolved cryo-EM methodologies, with a number of systems being reported, showing great promise in this area. However, although we and others have shown that TrEM can be used to follow biomolecular reactions at high resolution, there are limitations to the technique: only a limited range of concentrations are suitable for TrEM and a high particle number per micrograph is usually required to achieve high-resolution reconstruction. This is an important consideration because concentrations will affect reaction rates and TrEM requires relatively high concentrations compared with other biochemical methods. There is also a limited amount of data comparing TrEM experimental results with those from complementary techniques such as stopped-flow light-scattering measurements to date. Here, we chose the actomyosin system as a test system because its kinetics are well understood.

A graphical summary of the TrEM data is shown in Supplementary Fig. S3. The on-grid TrEM kinetics of actomyosin dissociation (7–640 ms) are in agreement with stopped-flow measurements, although we note that the buffer composition has been shown to affect the actomyosin dissociation rate and is not matched exactly between studies (White & Taylor, 1976 ▸; Millar & Geeves, 1983 ▸). There is a small but significant difference between the 340 ms on-grid and 400 ms in-flow time points (16% and 11% myosin bound, respectively; *p* < 0.0001, Mann–Whitney test). This may indicate that there is a small difference in mixing efficiency, with the in-flow mixer being more efficient.

The rebinding of myosin to F-actin is expected after most ATP has been hydrolysed, and is also observed in stopped-flow light-scattering measurements (White & Taylor, 1976 ▸). Published steady-state ATP turnover rates for skeletal myosin S1 (A1) are in the range 5–10 s^−1^, depending on the F-actin concentration and the buffer composition (Rosenfeld & Taylor, 1984 ▸). Given the fivefold excess of ATP in our TrEM experiments, our in-flow data agree well with previous studies. However, there is a substantial difference between the 640 ms on-grid and 700 ms in-flow time points (15% and 31% myosin bound, respectively; *p* < 0.0001, Mann–Whitney test). In part, this could be accounted for by laminar flow in the in-flow setup, with the in-flow reaction having a larger temporal spread, with residence times exceeding 2 s for ∼2% of particles. Other factors may also contribute to the difference between the 640 ms on-grid and 700 ms in-flow time points. For example, confinement of the reaction mixture on-grid could affect the reaction. The air–water interface has been shown to bind proteins quickly, so there is likely to be a competition between F-actin and the air–water interface for myosin binding. Moreover, binding to the air–water interface may cause protein unfolding and sequestering of the protein, creating an accumulation away from the bulk protein within the thin film layer on the grid.

**Figure 4:**
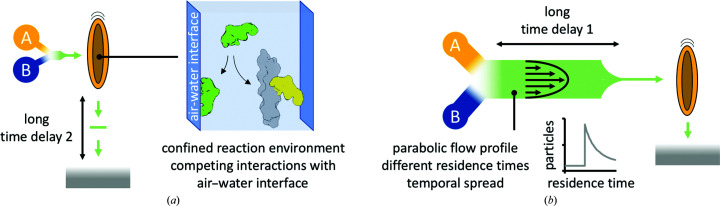
Graphical summary of challenges in TrEM. Schematics are analogous to Fig. 1 ▸(*a*). (*a*) The main time delay for on-grid mixing is the time between sample application and vitrification. During this time, especially for long time delays, the air–water interface can interfere with the reaction, for example by competing for binding. (*b*) For in-flow reactions, the main time delay is the time between mixing and sample application. In this case, the parabolic flow profile during laminar flow results in different residence times for individual molecules and a larger temporal spread.

From the data shown here, we conclude that there are important considerations when designing a TrEM experiment, which are summarized in Fig. 4 ▸. On-grid TrEM has the advantage that the time delay is minimally influenced by laminar flow. It can also allow the mixing of very different components that may not be suitable for microfluidic channels. However, when using this approach one factor which may need to be considered depending on the type and time frame of the reaction are interactions with the air–water interface, which may sequester proteins away and therefore alter the relative concentrations on-grid, potentially affecting the kinetics of the reaction or shifting the equilibrium (Fig. 4 ▸ *a*). This can be mitigated by the use of in-flow approaches, where mixing occurs away from the grid and therefore the time exposed to the grid and the air–water interface is low. A clear drawback of in-flow mixing is the temporal spread by laminar flow, which especially affects longer time delays (Fig. 4 ▸ *b* and Supplementary Fig. S3). However, it is preferable over on-grid mixing, particularly for even longer time delays than used in this study. On the time scale of seconds, evaporation, interactions with the air–water interface and/or interactions with the grid support or foil will have an even greater influence for on-grid mixing.

## Supplementary Material

EMDB reference: time-resolved cryo-EM structures of ATP-induced actomyosin dissociation, EMD-13328

Supplementary Figures. DOI: 10.1107/S2059798321008810/qn5004sup1.pdf

## Figures and Tables

**Table 1:** Conditions for actomyosin on-grid mixing experiments

	Flow rate (µl s^−1^)				
	Syringe 1 (actoymosin)	Syringe 2 (ATP)	Syringe 3 (ATP)	Spray–ethane distance (cm)	Plunge speed (m s^−1^)
7	2.08	1.04	1.04	1.4	2.0
13	1.04	0.52	0.52	2.0	1.6
340	2.08	1.04	1.04	4.9	N/A†
640	2.08	1.04	1.04	4.9	N/A†

† En route to vitrification, the grid was stopped and incubated for an additional 300 or 600 ms.

**Table 2:** Conditions for actomyosin in-flow mixing experiments

	Flow rate (µl s^−1^)			
Time delay (ms)	Syringe 1 (actoymosin)	Syringe 2 (ATP)	Delay line ID (µm)	Delay line length (cm)
400	2.08	2.08	150	2.0
700	2.08	2.08	360	2.0

**Table 3:** Data-collection and processing parameters

Data set	7 ms (on-grid)	13 ms (on-grid)	340 ms (on-grid)	640 ms (on-grid)	400 ms (in-flow)	700 ms (in-flow)
Data-collection parameters						
Detector	Falcon III	K2	Falcon III	Falcon III	Falcon III	Falcon III
Fluence (e^−^ Å^−2^)	72	52	72	62	62	62
Nominal pixel size (Å)	1.065	1.07	1.065	1.065	1.065	1.065
No. of frames	59	32	59	40	40	40
Processing parameters						
No. of micrographs	306	123	120	330	184	176
Original box size (pixels)	400	404	400	400	400	400
Initial No. of segments	89892	30070	46246	67198	20083	82353
No. of free actin segments (final)	27886	16617	32899	46513	13745	39044
No. of actomyosin particles (final)	31128	6495	5848	9686	2129	19270
