# Supplementary material for: On-grid and in-flow mixing for time-resolved cryo-EM
Source: Acta Crystallogr D Struct Biol. 2021 Sep 22;77(Pt 10):1233–40. doi: 10.1107/S2059798321008810 (PMC8489233; doi:10.1107/S2059798321008810)
Supplement: Supplementary file 1 [file d-77-01233-sup1.pdf]

STRUCTURAL
BIOLOGY

Volume 77 (2021)

Supporting information for article:

On-grid and in-flow mixing for time-resolved cryo-EM

David P. Klebl, Howard D. White, Frank Sobott and Stephen P. Muench

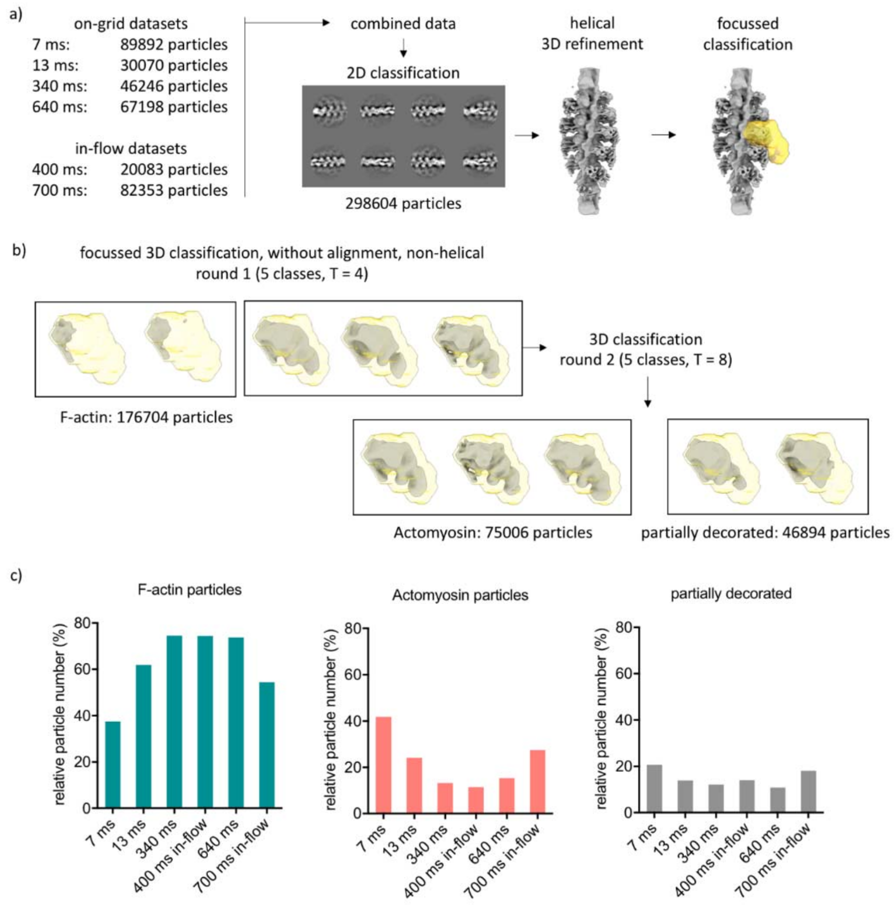

Figure S1 (a) All datasets were combined. One round of 2D classification was used to remove 'bad' particles. Helical refinement was then used to generate a consensus reconstruction with all 'good' particles from all timepoints. A myosin mask was generated that covered the central myosin binding site. (b) Focussed classification was used to separate F-actin and actomyosin particles. No helical constraints were used and particles were not aligned during classification. In two rounds of 3D classification, the dataset was split into F-actin, actomyosin and partially decorated particles. (c) Relative particle number for each class from each dataset. F-actin and actomyosin particles show a time-dependent change in particle number while partially decorated particles do not show as clear a trend.

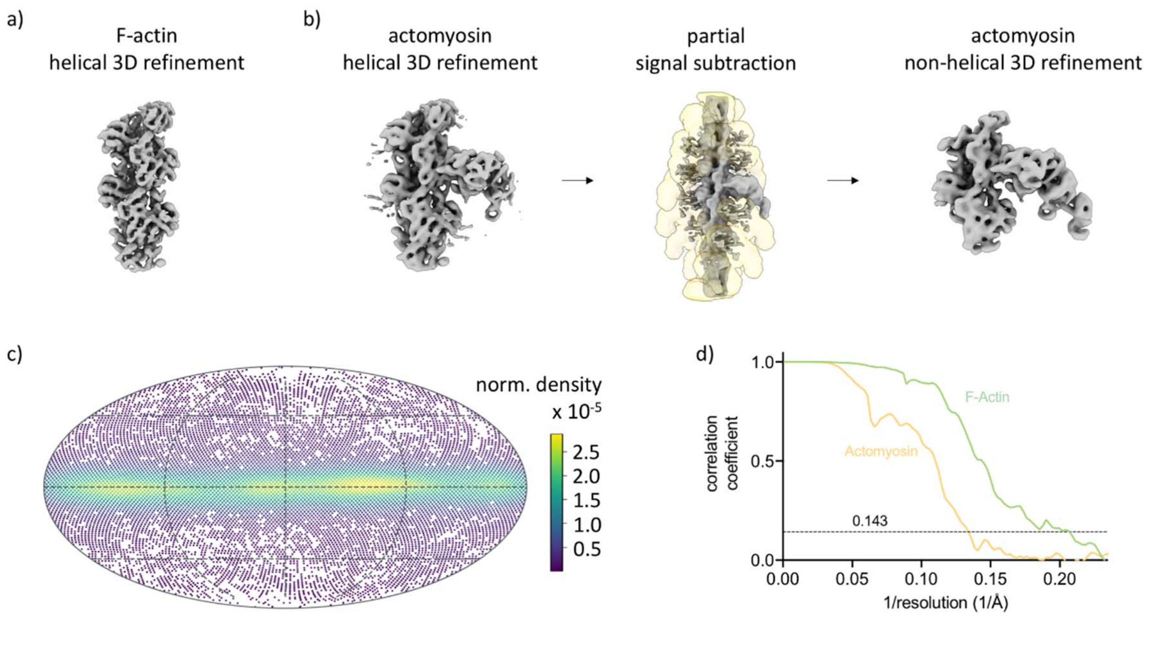

Figure S2 (a) Helical refinement of all F-actin subunits, shown is the sharpened final reconstruction. (b) Non-helical refinement used for actomyosin particles. A helical 3D reconstruction was made first, then a mask was created covering all but 3 central actin subunits and the central myosin binding site. After partial signal subtraction, a non-helical (single particle) reconstruction for the actomyosin particles was made. This shows improved density for the radially distant regions of the myosin. (c) Orientation distribution for the non-helical actomyosin reconstruction. Most particles show ~ 90 ° tilt angle (highest density along the equator), indicating that the angular assignment of most particles is sufficient without helical constraints. The colour indicates estimated normalised probability density. (d) Fourier shell correlation curves for the helical F-actin (green) and non-helical actomyosin (yellow) with the 0.143 threshold indicated as dashed line.

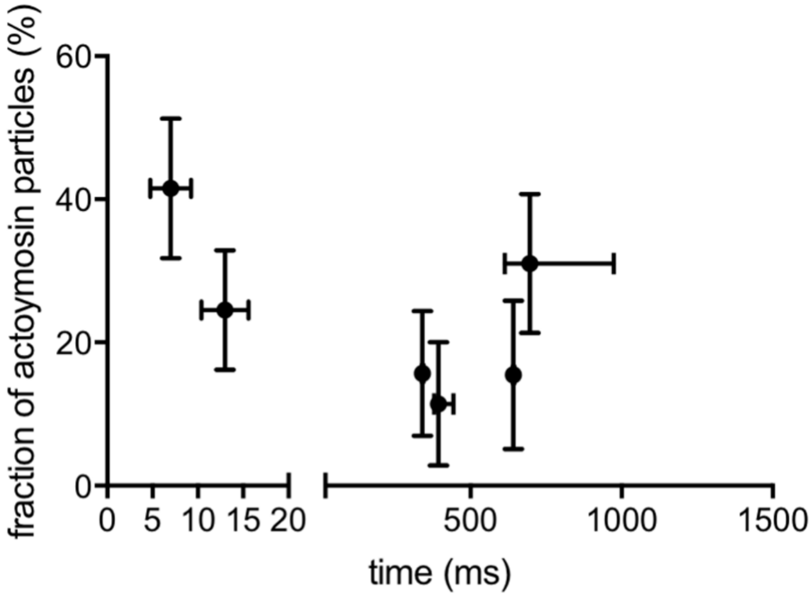

Figure S3 Plotted is the fraction of actomyosin particles determined by focussed 3D classification for each timepoint, shown are mean and standard deviation in y. For estimation of x error, see Methods section. For in-flow timepoints, the x value shown is the median and error bars in x represent 1 σ deviation in either direction as this better represents the skewed distribution.
